# Suicide Risk Among Persons with Dementia and Their Families: Insights from Population Mortality Registry

**DOI:** 10.1093/geroni/igaf122.4283

**Published:** 2025-12-31

**Authors:** Viktoryia Kalesnikava

**Affiliations:** Institute for Social Research, Ann Arbor, Michigan, United States

## Abstract

The onset and progression of Alzheimer’s and related dementias (ADRD) can impact the psychosocial well-being of persons with dementia and their families, increasing the risk of suicidal behavior, particularly among those diagnosed before age 65. While ADRD disproportionately affects racial and ethnic minorities and individuals from disadvantaged backgrounds, research on late-life suicide in persons with ADRD has been limited, especially among underrepresented groups. This study examined individual and contextual suicide correlates among persons with ADRD and their relatives, applying large language models to structured and textual data from the National Violent Death Reporting System (2003-2022). Among suicide decedents aged 45 yo or older, 7654 deaths were linked to ADRD: 51% had confirmed ADRD, 34% had MCI/suspected ADRD, and 14% were relatives of persons with ADRD. Decedents with confirmed ADRD had higher odds of traumatic brain injury (TBI), schizophrenia diagnosis and were twice as likely to die in a long-term care facility compared to age-, sex-, and US State-matched non-ADRD decedents. Decedents with MCI/suspected ADRD more often had college education or beyond and higher odds of depressed mood, anxiety, PTSD, and TBI. Decedent relatives of persons with ADRD were more often married, had higher odds of experiencing family or relationship stress and housing instability, but were less likely to be from racial or ethnic minority groups or have lower education. Our study describes variations in potentially modifiable late-life suicide risk factors across ADRD-related groups, highlighting the need for tailored psychosocial support for both persons with ADRD diagnosis and their families.

